# Risk factors for hepatitis C virus sero-positivity among haemodialysis patients receiving care at Kidney Centre in a tertiary health facility in Maiduguri, Nigeria

**DOI:** 10.11604/pamj.2014.19.305.5639

**Published:** 2014-11-21

**Authors:** Ibrahim Ummate, Ballah Akawu Denue, Ibrahim Musa Kida, Onah Joseph Ohioma, David Bukbuk Baba, Waru Goni

**Affiliations:** 1 Department of Medicine, Nephrology Unit, PMB 1414, University of Maiduguri, Teaching Hospital, Borno State, Nigeria; 2 Department of Medicine, Infectious Disease and Immunology Unit, PMB 1414, University of Maiduguri, Teaching Hospital, Borno State, Nigeria; 3 Field Researcher, Bill Gates Foundation, Abuja, Nigeria; 4 Department of Microbiology, PMB 1069, University of Maiduguri, Borno State, Nigeria

**Keywords:** HCV, haemodialysis, risk factor, transfusion

## Abstract

Hepatitis C virus (HCV) is an important health care problem in haemodialysis. Hepatitis C virus is both a cause and complication of kidney diseases. Yet there are limited information on antibody against HCV in patients on haemodialysis. The purpose of this study was to determine the prevalence of anti-HCV and the risk factors associated with HCV infection in a cohort of 100 participants on haemodialysis. They were consecutively recruited into the study, anti-HCV testing was made by the 3rd-generation ELISA System (C-100, C-33c, C-22). The prevalence of HCV antibody was 15%, risk factors associated with HCV antibody were history of blood transfusion and duration of session of haemodialysis; the risk increased with increased with the number of blood transfusion and seasons of haemodialysis. The observed high prevalence of HCV antibody among patients on haemodialysis reflect the quality of healthcare services and the standards of infection control practices in our haemodialysis units. Routine screening for HCV should be done before blood transfusion using third generation ELISA assays with high sensitivity and specificity. Safety measures should be taken in our haemodialysis units to prevent cross infection among patients and staffs. These safety measures include; discarding syringes, needles, gloves, bloodlines and dialysers after single use, and the use of sterile dressings on each patient visit.

## Introduction

Hepatitis C virus (HCV) is a significant cause of morbidity and mortality among chronic renal failure patients due to their inability to clear the virus efficiently [1]. Patients on haemodialysis dependantant on blood transfusion instead of erythropoietin to reverse anemia are at particular risk of acquiring HCV as it is easily transmissible through blood and blood product [2–4]. Other factors reported to favour HCV acquisition among patients on dialysis include cross infections from the sharing of dialysis machines and the dialysis equipment, the reprocessing of dialyzers and blood lines and the increased requirement of blood transfusions [5, 6]. Studies have reported a significant association between the dialytic age and anti-HCV positivity; dialytic age has been shown to be predictor for the risk of the acquisition of the HCV infection [5–7]. Furthermore, although repeated dialysis increases the risk of contracting HCV, there is no risk through the equipment used in dialysis [8]. The prevalence of HCV is less prevalent in developed countries due to socioeconomic factors, better infection control measures, use of erythropoietin instead of blood transfusion to treat anemia [9]. Conversely the prevalence of hepatitis C virus infection is expected to be high in developing countries especially in patients with chronic kidney disease because of exposure of these individuals to multiple risk factors such as blood transfusion and haemodialysis. Unfortunately information on the risk factors of HCV among renal failure patients is sparse in Nigeria. To the best of our knowledge, no study has reported the risk factors associated with HCV acquisition in our environment. We therefore set out to determine the risk factors associated with contracting HCV among patients on Haemodialysis receiving care at a kidney centre attached to tertiary health institution in Maiduguri, Nigeria.

## Methods

This cross sectional analytic study considered one hundred consecutive patients with stage 5 chronic kidney failure. Participants were either recruited at kidney centre or on medical wards of the University of Maiduguri Teaching Hospital. Patients were subsequently examined. Patient's demographic data including age and sex, were recorded. Risk factors, clinical features, possible aetiology of chronic kidney failure, and laboratory data were obtained by the use of questionnaire. Blood samples were obtained at entry for creatinine clearance, serum electrolytes, urea, and creatinine, including serum calcium and phosphate, liver function tests, HIV screening, Anti-HCV testing was made by the 3^rd^ generation ELISA System (C-100, C-33c, C-22), HbsAg and full blood count (FBC). Abdominal ultrasound scan was also done on all the patients. Case definition of patients with stage 5 chronic Kidney failure i.e eGFR <15 ml/min was based on the estimated glomerular filtration rate (eGFR) by Cockroft-Gault equation [10]. All data were collected and statistical analysis was performed using Epi Info 2002. Chi-square test & Fisher's exact test were used for comparing categorical variables. A probability of less than 0.05 was considered statistically significant. Permission to conduct this study was obtained from research and ethics committee of University of Maiduguri Teaching Hospital. Informed consent was obtained from all patients.

## Results

### Sociodemographic characteristics

**Figure 1:**
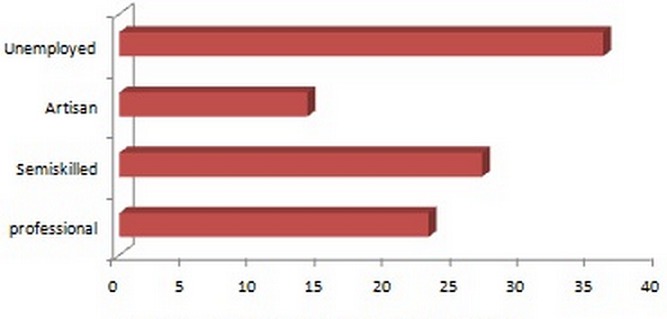
Proportion (%) of participants by occupation

**Figure 2:**
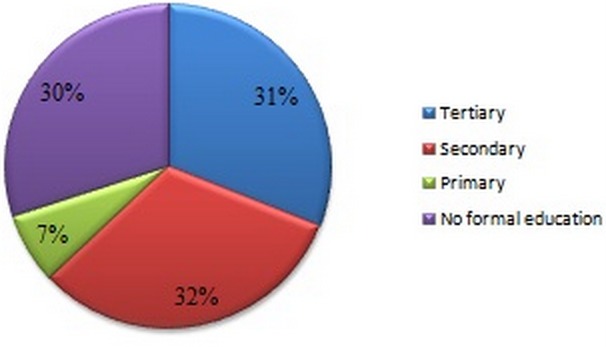
Distribution of participants by educational status

The participants consisted of 68 (68%) males and 32(32%) females, with most of them in 3rd and 4^th^ decade of life. Their ages ranged between 15 and 74 years with a mean (± SD) of 39.9 ± 13.58 years. Males with mean age (± SD) of 41.71 ± 13.27 were older than females that had mean age (± SD) of 36.06 ± 13.64 years. Majority of the participants, 79 (79%) were in stable marriage union, while 21(21%) were single. The age and sex distribution of the studied participants is as depicted in Table 1. Most of the patients (32%) had secondary education followed by tertiary education in (31%). Thirty patients (30%) had non-formal education whereas 7 (7%) had primary education. Figure 1 shows the educational status of the patients. Majority of the study subjects (36%) were unemployed. Semiskilled workers followed with 27%. Twenty-three percent of the study patients were professionals while 14% were artisans as shown in Figure 2.

**Table 1:** Age and sex distribution

Age (years)	males, no(%)	females, no(%)	Both, no(%)
≥19	03(3%)	03(3%)	06(6%)
20-29	09(9%)	08(8%)	17(17%)
30-39	17(17%)	07(8%)	24(24%)
40-49	20(20%)	09(9%)	29(29%)
50-59	11(11%)	03(3%)	14(14%)
60-69	06(6%)	02(2%)	08(08%)
≥70	02(2%)	-	02(02%)
Total	68(68%)	32(32%)	100(100%)

### Risk factor for hepatitis C virus infection

We sought to determine the risk factors for HCV infection in our study population. The prevalence of HCV among our studied cohort was 15 (15%). Fifty five (55%) of the patients had no identified risk factor for HCV infection. A total of 33 (33%) patients had received blood transfusion, 17 (17%) of these patients had blood transfusions alone; 15 (15%) had received blood transfusions and were on haemodialysis as well. One patient was a haemophilic and had received blood transfusions. Of the 33 patients who received blood transfusion, 10 (10%) tested positive for HCV antibodies. When taking into consideration the 33 patients who received blood transfusion, it could be seen that 26 patients received 1-5 units of blood and among whom 3 patients were positive for HCV abs. While the 2 patients who received 6-10 units of blood and the 5 patients who received =11 units of blood were all positive for HCV abs. Blood transfusion therefore was found to be an important risk factor for HCV infection with a p value of 0.0000. Taking the risk of HCV through haemodialysis into account, patient that had ≥ 10 sessions were all negative for HCV abs, while the 2 patients who had ≤11 sessions were positive for HCV antibody. Haemodialysis was also found to be a risk factor for HCV infection with a p value of 0.0095. Evaluation of the risk factors of HCV antibody is as presented in Table 2.

**Table 2:** Risk factor for HCV among the participants

Risk factor	HCV negative	HCV positive
*Blood transfusion*		
1-5 units	23	03
6-10 units	-	02
≥11 units	-	05
Total	23	10
**Fisher's exact test, p = 0.0000**		
*Haemodialysis*		
1-5 sessions	12	-
6-10 sessions	01	-
≥11 sessions	-	02
Total	13	02
**Fisher's exact test, p = 0.0095**		
*Health worker*		
1-10 years	03	-
10-20 years	01	-
≥20 years	-	02
Total	04	02
**Fisher's exact test, p = 0.07**		
*Intravenous drug abuse*		
1-5 years	03	-
6-10 years	-	01
≥ 11 years	-	02
Total	03	03
Fisher's exact test, p = 0.10		

## Discussion

The point prevalence of HCV antibody of 15% among patients on haemodialysis in this study is alarmingly high in comparision to 1.4% among prospective blood donors earlier reported in our environment [11]. The observed high prevalence of HCV infection among the patients on haemodialysis reflect the quality of healthcare services and the standards of infection control practices in haemodialysis unit. The prevalence among blood donors may be presumed to reflect the general population as they are exposed to similar risk as the general population. Several studies have identified transfusion of blood and blood product as a significant risk factor for acquisition of HCV [3–7]. This proposition was corroborated in this report as we established blood transfusion as a major factor for HCV (p = 0.000). We also observed the risk to linearly increase with the number of transfusion (p =0.000). This is not surprising, as those who received multiple blood transfusions were more likely to receive transfusion of unscreened blood or to receive blood transfusion from centres that don't have facilities for HCV antibody detection, as such are likely to be positive. Conversely other studies documented that the number of transfusions did not correlate with the anti-HCV prevalence [8, 9, 12] which may be due, among other factors, to a bias of data collected on the number of transfusions and safety standard [8]. Although the mode of transmission of HCV in dialysis is yet to be fully elucidated. We identified association between increased sessions of haemodialysis with risk of HCV (p = 0.0000). Studies have shown that contamination of the ultrafltrate, fluid that is removed from the blood during the dialysis procedure might constitute a potential risk for HCV transmission. It is possible that HCV infection could be transmitted from patient to patient in haemodialysis units, infact epidemics have been reported in some haemodialysis units. Also a breach of the safety devices on the haemodialysis machine could lead to transmission of infection [6–9, 13, 14]. Health profession exposes hospital staff to various infections particularly those that have direct access to patients, body fluids and equipment. Studies by Thorburn et al [15], Thomas et al [16] and Neal et al [17] found health profession to be an important risk factor for HCV infection. We are unable to validate or refute this finding due to limited health workers in our study.

### Limitations

Although we detected antibody against HCV using third generation ELISA assays with high sensitivity and specificity. We were unable to detect and estimate HCV-RNA using reverse transcription-polymerase chain reaction (RT-PCR).

## Conclusion

The observed high prevalence of HCV antibody among patients on haemodialysis reflect the quality of healthcare services and the standards of infection control practices in our haemodialysis unit. Routine screening for HCV should be done before blood transfusion using third generation ELISA assays with high sensitivity and specificity. Safety measures should be taken in our haemodialysis units to prevent cross infection among patients and staffs. These safety measures include; discarding syringes, needles, gloves, bloodlines and dialysers after single use, and the use of sterile dressings on each patient visit.
